# Testing Emotional Eating Style in Relation to Willingness to Pay for Nutritional Claims

**DOI:** 10.3390/nu11081773

**Published:** 2019-08-01

**Authors:** Belinda López-Galán, Tiziana de-Magistris

**Affiliations:** 1 Centro de Investigación y Tecnología Agroalimentaria de Aragón (CITA), Unidad de Economía Agroalimentaria y de los Recursos Naturales, Saragossa 50013, Spain; 2 Instituto Agroalimentario de Aragón (IA2), CITA-Universidad de Zaragoza, Saragossa 50059, Spain

**Keywords:** emotional eating, food choices, nutritional claims

## Abstract

In face of the high prevalence of non-communicable diseases, nutritional claims represent a useful tool to help people to make healthier food choices. However, recent research notes that when some people experience an intense emotional state, they increase their food consumption, particularly of energy-dense and sweet foods. In consequence, this study aims to assess whether emotional eating (EE) style influences the purchase of food products carrying these claims. To this end, a real choice experiment (RCE) was conducted with 306 participants who were asked to evaluate different types of toast. An error component random parameter logit (ECRPL) was used to analyze their preferences for reduced-fat and low-salt claims toast and the effects of the variation of the EE score on individual preferences. Findings of this study suggest that emotional eating negatively impacts purchasing behavior related to nutritional claims. In particular, a decrease of the willingness to pay between 9% and 16% for every unit of toast with nutritional claims was noted when an increase of EE individual score was registered. In this regard, to increase the effectiveness of the nutritional claims, policymakers and private sectors should consider the management of individuals’ emotional states in designing public health policies and marketing strategies, respectively.

## 1. Introduction

The increase in non-communicable diseases (NCDs) is related to the poor quality of the human diet [1]. The World Health Organization (WHO) reports that cardiovascular diseases, cancer, respiratory diseases, and type II diabetes are the main causes of premature death for over 40 million people worldwide [2]. This poor state of public health [2] has had a negative impact on public health expenditure [3,4] and labor productivity [5]. As a consequence, policymakers have designed instruments to help people make better food choices [6]. 

One such instrument is the nutritional claim [7], a statement informing consumers if a food product has been reformulated through reduction or addition of one or more nutrients (e.g., reduced fat, low salt, high fiber) [7]. This reformulation must be in line with scientific evidence confirming that high consumption of a specific nutrient can be harmful to health (e.g., sugar or fat) or, conversely, that the food product contains a nutrient that is vital to a healthy diet (e.g., fiber). Hence, the objective of policymakers is to improve consumers’ decision-making by increasing their awareness through the provision of scientifically proven nutritional information, thereby reducing the effects of the poor nutritional knowledge or impulsivity [7].

Preference studies suggest that nutritional claims are useful to consumers in choosing food products [8,9,10,11,12]. However, Prieto-Castillo et al. [13] calculated that, while 54% of Spanish consumers seem to understand nutritional labeling, only 20% use it in their decision-making. In this regard, evidence suggests that personal factors such as familiarity with the claim [8], taste [14], certificated quality [11], health concerns from past events [9,12], and psychological factors—such as body image [15] and emotional intelligence [16]—all influence the purchase of food products with nutritional claims. 

In general, the influence of emotions on eating has been well-studied in terms of the behavioral [17,18] and the physiologic [19] components. However, this effect is too complex to predict due to the variability of emotional intensity among individuals [20]. Despite this, there is general consensus in research that emotions can negatively influence eating behavior when individuals present either poor ability to manage their emotions or high desire for eating [21]. 

**Figure 1:**
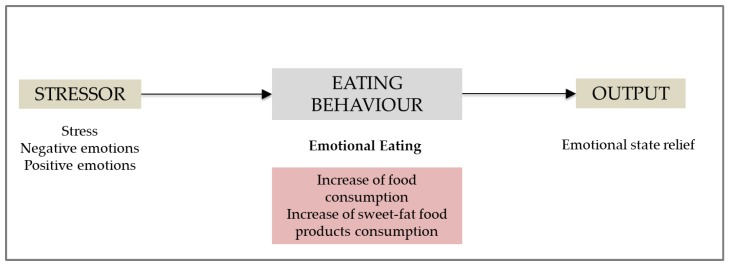
Emotional eating mechanism.

Behavioral approaches identify a consistent construct that distinguishes people who eat more in response to emotions [22] because they are cue-reactive to external stimuli and those who do so when they cannot manage their emotions [21]. The latter behavior is known as “emotional eating”, characterized by an overconsumption of high-density foods as a coping strategy to stress [23] or intense emotions, both negative [22] and positive [24]. Emotional eaters seem to display this pattern when an stressor appears in their everyday life, triggering an increase in food consumption, particularly that of sweet-fat (i.e., energy-dense) food products, leading to an improvement in their emotional state (Figure 1) [22,24]. 

Studies that found that high levels of stress appear to modify existing eating behavior [23,25,26,27,28]. For example, Roberts [23] noted that healthy women increase their consumption of saturated fat (26%), carbohydrates (30%), and alcohol (33%) when they experience a significant increase in cortisol secretion (a physiologic parameter of stress). Groez et al. [27] indicated that perceived stress in women—irrespective of body mass index (BMI)—relates to a significant decrease in healthy food consumption and an increase in consumption of chips, hamburgers, and soft drinks. 

Negative emotions are another stressor relevant to emotional eating behavior [29,30,31,32,33]. Van Strien et al. [30] reported that when sadness was induced in women, those who scored high on emotional eating increased their consumption of snacks. Additionally, the authors suggested that these participants use their eating behavior as a coping strategy to manage their emotional state. In another study, Van Strien et al. [33] noted that low emotional eaters did not increase their food intake when they experienced sadness or happiness, but high emotional eaters did so after the induction of a negative or positive emotional state. Only sweet foods present an increase of consumption. 

Although most studies tie the overconsumption of energy-dense food products to negative emotions, recent research demonstrated that inducing positive emotions can also increase food consumption and/or that food intake improves people’s emotional state [24,34]. In particular, Bongers et al. [24] found that self-reported female emotional eaters consumed more hedonic food (i.e., chips and chocolate) when they experienced positive emotions compared to neutral ones. The meta-analysis of Cardi et al. [34] similarly concluded that positive mood induced an increase in the eating of both sweet and savory foods. In addition, even healthy people can present emotional eating behaviors in social events that highlight the hedonic nature of their food intake. 

However, some studies did not find negative or positive emotions to trigger emotional eating behavior [21,35,36]. Bongers et al. [35] found that negative emotions do not determine increase or decrease in appetite, and they consider negative and neutral emotional states to have equal probability in influencing appetite. Evers et al.’s [36] meta-analysis did not find any impact of negative or positive emotions on the consumption behaviors of self-reported emotional eaters. The authors agreed with Bongers et al. [21] that one reason for these results is that scales used to identify emotional eaters do not properly capture changes in their eating behavior.

Despite this evidence, Evers et al. [36] and Cardi et al. [34] reported great heterogeneity in the existing research findings, which they included in their meta-analyses. They noted that this could be explained by differences in the emotion induction procedure, sociodemographic characteristics of the samples, types of food offered, or the emotional eating scales used in the studies. In light of this information, the main results expected in the present study suggest that emotional eating style has an impact on purchasing behavior. 

Moreover, evidence suggests that emotional eaters are more likely to develop eating disorders, such as binge eating [37] and anorexia nervosa [38]. These individuals have a high risk of weight gain as well as difficulty maintaining or losing weight [39]; they also present some personality traits such as neuroticism [29] and impulsivity [35]. Hence, since (a) food choices involve more than simple intake, (b) food purchase is the preliminary step in the decision-making process towards healthy or unhealthy intake [40], and (c) purchasing decisions share an impulsivity component with intake [41], it seems logical to suppose that, if emotional eating influences consumers’ eating behavior in terms of intake, the same may occur when they are shopping and paying for food products. 

In light of the above information, this study mainly aims to assess whether the emotional eating style has an influence (positive or negative) on consumer preferences for a food product (e.g., toasted bread) with nutritional claims (e.g., reduced-fat and low-salt). To achieve this objective, we conducted a real choice experiment (RCE), which is the most widely used stated preference multi-attribute method for valuing products or attributes. The results of the study contribute towards understanding individuals’ psychological factors, which could be taken into consideration by stakeholders to improve the efficacy of public health policies or intervention programs. 

## 2. Materials and Methods

### 2.1. Real Choice Experiment

This study follows the premise of non-hypothetical methods. An RCE, which is considered an incentive-compatible mechanism, was conducted with a real food product. The choice experiment (CE) is currently the most widely used stated preference method to evaluate consumer demand for market and nonmarket products. It is consistent with Lancaster’s consumer theory [42] and random utility theory [43]. The main advantage of choice modeling against other valuation methods (e.g., contingent valuation, experimental auction) is the similarity of the choice task to actual consumer purchase situations, such as decisions made in supermarkets. For example, respondents are presented with several alternatives of a product, each with different attributes and levels in a series of multiple-choice tasks. In each task, they are required to choose which, if any, of the offered items they would purchase.

However, one main critique against this method is the existence of hypothetical bias, defined as the difference between the values obtained through hypothetical methods and those gathered through non-hypothetical approaches (or what an individual might actually pay for the good) [44,45]. Hence, participants may overstate their willingness to pay (WTP) in hypothetical settings and behave inconsistently when they do not have to support their choices through real financial commitments [46,47].

To mitigate this bias, the RCE was used. This is also its main advantage. The choice of methodology ensures that preferences are truthfully revealed and that estimated WTP values are real. Studies conducted by Chang et al. [48], Loomis et al., [49], Lusk and Shogren [50], Grebitus et al. [51], and de-Magistris et al. [52] confirmed that non-hypothetical choices are better approximations of true preferences than hypothetical ones based on comparisons of the hypothetical CE with the RCE and with actual market shares. Furthermore, since the final samples of the studies represent the population according to sociodemographic profiles, the estimated WTP can be considered representative of the Spanish population. This methodology allows estimation of consumer WTP for nutritional claims, which can be considered actual payments in the marketplace at the time of the experiment.

### 2.2. Recruitment and RCE Procedure

The study was conducted in March and April of 2015 in the capital town of Saragossa, Spain. As the city’s size, sociodemographic characteristics, and level of income are representative of the country as a whole, the main results can be extrapolated to the Spanish population [53,54]. 

A total of 306 individuals were recruited for the experiment by a subcontracted professional market research agency using a stratified sampling procedure by gender, age, level of study, and BMI. The sample size resulted in a sample error of +/− 7% and a confidence level of 95.5% (K = 2) when estimating proportion (p = q = 0.5). The a priori power calculation analysis resulted in a medium effect size of d = 0.80 with α = 0.05 and a power of 0.80, but the post-hoc analysis of the sample resulted in 0.995 [55]. The market research agency selected legal-age participants (18 years or older) who were primarily food buyers in households that consume toast. The experiment followed the guidelines in the Declaration of Helsinki, and the protocol was approved by the Ethics Committee of CITA (FP7-MC-CIG-332769).

Participants were divided into groups of 10 to 12. During the experiment, they were seated separately from one another to avoid any communication between them. Each session was conducted as follows. Before beginning, all the participants signed a consent form for their inclusion in the study. They were then informed that the experiment was structured in two parts, a questionnaire and a choice task. First, they filled in the questionnaire, which included the emotional eating scale (see Section 2.4.1 for more details) and items on their sociodemographic characteristics. After completing this, they were informed about the aim of the experiment, the methodology, and the food products used for the analysis. They were also notified that they would receive €10 in cash as a participation fee at the end of the session; this could be used to buy one of the products presented in the experiment.

The participants were allowed to inspect all the toast packages (which included the nutritional claims) on the designated shelf of the experiment room and which corresponded to products present in the Spanish market. During this inspection, they could find information related to the type of toast, nutritional claims, and price per package, but information about brands, ingredients, and manufacturing method was missing.

Afterwards, the researcher explained that the shelf contained 12 choice sets, which included three options: two packages of toast and a no-buy option. In addition, after checking each set, participants were required to write down their choices (one of the two items or no purchase) on a sheet given to them at the beginning of the session. Finally, the researcher instructed them to write a number from 1 to 12 (corresponding to each choice set) on another piece of paper, and she then selected one of them from an envelope to determine the binding choice set. If a participant chose any of the toast options, the researcher had to charge the indicated price (deducted from the €10 reimbursement) and give him or her the selected item. However, if the no-buy option was selected, the participant received the full €10.

### 2.3. Product and CE Design

A food product category representative of the Spanish diet was selected to analyze consumers’ preferences in nutritional claims according to their eating styles. In particular, a package of 270 g of toast was selected, because it is essentially a cereal. Cereals are the principal food group that contributes to total energy intake (25%) in the Spanish diet [56], and bread is the main product in this category [56]. Moreover, toasted bread is one of the most commonly consumed types of bread in Spain (10%) [57].

Table 1 presents the selected attributes and levels used in the RCE. Three attributes were selected: price and two nutritional claims. To include an extended price range, four levels were considered (€0.70, €1.15, €1.60, and €2.05); these were set based on the prices in the Spanish supermarket at the time of the experiment. A reduced-fat claim (FAT), a low-salt content claim (SALT), and an interaction between both claims (FSALT) were used to identify consumer preferences regarding nutritional information.

Two levels of nutritional claims were considered. The first, unlabeled condition represented the conventional toast option that did not carry any claims. The second level contained a nutritional claim. The FAT option indicated that the bread was produced with a 30% reduction in fat compared to traditional toast. Additionally, this claim was selected because consumers perceive bread to be a fattening food and tend to eliminate it from their diet [58]. On the other hand, it is well-known that high consumption of fat is related to the prevalence of non-communicable diseases such as cardiovascular illnesses and obesity [59,60]. Finally, the SALT claim indicated that the bread did not contain more than 0.03 g of salt per 100 g. This option was considered because, according to the WHO [61], consumption of salt is a risk factor in kidney disease, osteoporosis, and high blood pressure [62,63], and intake should be reduced to 5 g per day.

**Figure 2:**
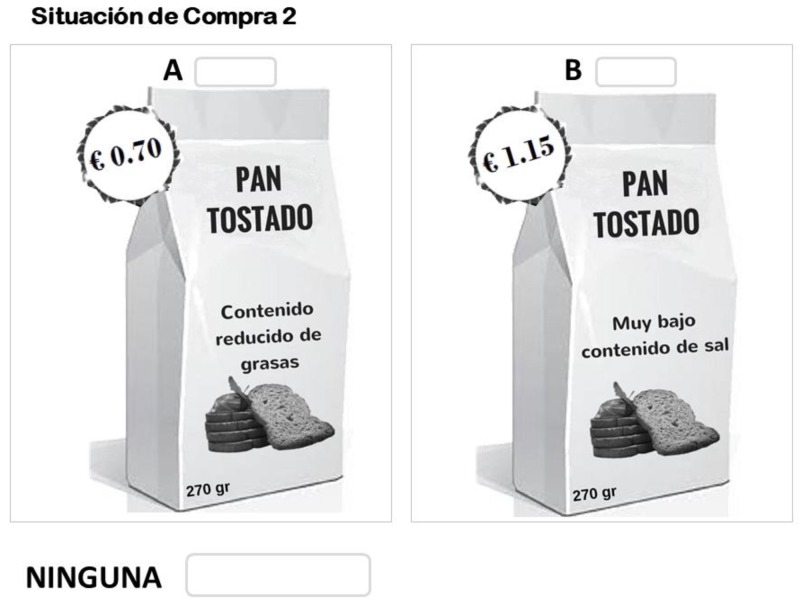
Example of a choice set.

To reduce the hypothetical bias, a sequential Bayesian approach was used to minimize D-error. This design was selected because the parameters estimated (which measure taste preference for each nutritional claims separately) present the lowest possible standard error; consequently, the design can be considered efficient [64,65]. Following Scarpa and colleagues [65], three steps were performed to determine the choice task. First, a pilot study was conducted to specify the basic multinomial logit (MNL) model. Second, an orthogonal factorial design was created with the selected attributes and levels in the first step. Third, a database of the pilot study was used to establish a model whose estimated coefficients served as Bayesian priors. Finally, the last model indicated that a choice design with 12 tasks was needed to obtain an efficiency of 96.6%. Moreover, each choice task was required to contain three alternatives—two consisting of different options of toast and a no-buy option (Figure 2). Ngene software version 1.1.2 [66] was used to obtain the choice design.

### 2.4. Measures

#### 2.4.1. Emotional Eater Questionnaire

Emotional eating was measured using the Spanish version of the emotional eater questionnaire (EEQ) proposed by Garaulet and colleagues [67]. This scale identifies the relationship between negative emotions and food intake in an individual. It is composed of ten items rated on a four-point system (never, sometimes, generally, and always). The total score is obtained by summing all the items; “never” corresponds to 1 point and “always” corresponds to a score of 4. Lower scores indicate that the individual exhibits a good relationship between emotions and food intake, therefore their eating behavior is healthy. Appendix A presents the English and Spanish version of the questionnaire.

Moreover, the EEQ enables classification of the sample into four groups based on total score. The first category is the non-emotional eaters (scores between 0 and 5). This group has stable eating behaviors; they decide to eat or not according to their hunger cues and not their emotions. The second group is the low emotional eaters (6 to 10); although their eating behavior is not based on emotions, they find certain foods irresistible. The third group is the emotional eaters (11 to 20); certain moods moderate how and how much they eat, but they are generally in control of their eating patterns. The last category, the very emotional eaters (scores between 21 and 30) present the least healthy eating behavior, where their moods moderate their food intake—these individuals are at high risk of developing eating disorders.

The Cronbach’s alpha of the total scale was calculated to assess the adequacy of the EEQ in the sample. The result, 0.86, is a high score in this reliability index.

#### 2.4.2. Model Specification

Choice experiments were developed following the Lancaster [42] and the random utility theories [43]. The former supposes that the utility of a food product can be broken down into the utility of its component attributes, while the latter suggests that its utility is clear to the individuals but not to the researcher. The researcher can only observe some attributes but not the components of the individual utility, which is treated as stochastic. Utility is considered a random variable obtained from the *n*th individual facing a choice among *j* alternatives in one of *t* choice occasions; it can be represented as follows:

(1) $$U_{njt}=\beta x_{njt}+\varepsilon_{njt}$$

Here, *β* is a vector representing the estimated parameter, and *ε_njt_* is a random term that follows an extreme value type I (Gumbel) distribution; the latter is also the independent identically distributed (iid) error term over time, people, and alternatives. Hence, given the attribute and the respective levels used in this study, the utility function becomes:

(2) $$U_{njt}=\mathrm{ASC}+\beta_{1\;}PRICE_{njt}+\beta_{2\;}FAT_{njt}+\beta_{3\;}SALT_{njt}+\beta_{4\;}FSALT_{njt}+\varepsilon_{njt}$$

ASC is an alternative-specific constant, coded as a dummy variable equal to 1 for the no-buy option and 0 otherwise. The price (PRICE) is represented in the model as a continuous variable. The claims *FAT*, *SALT,* and *FSALT* are coded as dummy variables where a value of 1 means that the corresponding claim is present on the toast package and 0 indicates that it is absent.

To assess preferences, various assumptions can be considered. First, consumers tend to choose the option that provides the highest utility from those available. Empirical evidence has demonstrated that consumer preferences are heterogeneous instead of homogeneous [15,68,69]. Hence, to obtain the mean and the standard deviation of each random parameter, it is necessary to consider a structured data panel to allow the next assumption that each individual made several choices, i.e., 12 in the case of the present study [70].

Consequently, a parameter logit model (RPL) could be established. However, this model assumes that the two toast options are correlated with each other and the no-buy option (ASC) is constant across the choice tasks. It supposes that the correlation between the two buy options would be higher than the no-buy option—in other words, the three alternatives share an extra error component. Thus, an error component random parameter logit (ECRPL) model may be used to relax this assumption and account for the extra error component missing in the utility function [64]. At this point, although emotional eating style can explain the heterogeneity in consumer preferences, it would be difficult to confirm that the former causes the latter.

Additionally, the RPL model also assumes that taste parameters are independent of one another; however, some attributes of the food product are likely interrelated. Thus, a Cholesky matrix could determine this interdependence in the taste parameter utilities of toast [71]. With this new assumption, an ECRPL model with emotional eating style interaction could be estimated.

As the aim of this study is to analyze whether emotional eating style explains heterogeneity in taste preferences regarding nutritional claims, EE was introduced in the model as a continuous variable. Following Bazzani et al. [72], before this introduction, the total score of an individual was mean-centered to allow for not only the marginal utility estimation of each nutritional claim but also the influence of emotional eating style on heterogeneity. In particular, the model reveals how variation from the mean value of the emotional eating score affects individual preferences for reduced-fat and low-salt content claims on toast.

The utility function is transformed to include the EE effects [Equation (3)]:

(3) $$\begin{array}{c} U_{njt}=\mathrm{ASC}+\beta_{1\;}PRICE_{njt}+\beta_{2\;}FAT_{njt}+\beta_{3\;}SALT_{njt}+\beta_{4\;}FSALT_{njt}+\beta_{5\;}FAT_{jt}\times EE_{n}\\ +\beta_{6\;}SALT_{jt}\;\times EE_{n}+\beta_{7\;}FSALT_{jt}\;\times EE_{n}\;+\;1_{j}\eta_{nt}+\varepsilon_{njt} \end{array}$$

In the above formula, β_5,_ β_6_, and β_7_ represent the respective coefficients of the interaction term between the attributes FAT, SALT, and FSALT with the emotional eating scale. As in Equation (2), the attributes are assumed to be random, while PRICE, ASC, and the interaction term variables are considered fixed. The *1_j_ŋ_nt_* term represents all the alternatives of the toast package design (*1_j_)* as well as the error component associated with only the two alternatives of the product and not the no-buy option (*ŋ_nt_*) [73]

To assess the robustness and the consistency of the model and confirm that the estimated taste parameters were in line with actual purchasing behavior, changes in the Akaike information criterion (AIC) and the log likelihood (LL) were considered. Lower values of both indicate that the model is a better fit. Nlogit software version 6 was used to performer the statistical analysis and to estimate the final econometric model. 

## 3. Results

### 3.1. Descriptive Analysis of Sociodemographic Characteristics

Three hundred and six individuals participated in the CE, and their sociodemographic characteristics are presented in Table 2. The majority of the sociodemographic characteristics of the sample did not show a significant statistical difference from the characteristics of the Spanish population. However, a slight majority were females (60%), who were overrepresented in comparison to the Spanish population. The average age of the sample was 45—two years older than the mean age of the nation. In this regard, 41% of the participants were middle-aged (35 to 55 years old). Most completed secondary studies (43%) and had household incomes between €1501 and €2500. The average BMI of the sample was 26, meaning than the participants were generally overweight, although the majority percentage of the sample were of normal weight (48%). The mean score on the emotional eating scale is 12, thus the sample can be classified as emotional eaters. In fact, 50% of the participants were rated to have an emotional eating style.

### 3.2. Nutritional Claim Preferences Regarding Emotional Eating

Table 3 reports the estimated results for the models specified in Section 2.4.2. The first (model 1) is the basic RPL, which assumes heterogeneity in consumer preferences. Model 2 incorporates an error component into the utility function to account for correlation between the alternatives, and model 3 makes the same assumption but considers the interaction between utility and EE style.

For the estimation, 500 Halton draws were used instead of pseudo-random ones to provide a more accurate simulation for the econometric model specification.

The LL, the AIC, and the AIC/N of each model were compared to identify which of the three models presents the best fit. In this regard, the LL improved noticeably from models 1 to 2 and slightly more from 2 to 3. On the other hand, the AIC and the AIC/N decreased from models 1 to 2 and then stabilized for the subsequent models. This improvement in fit from model to model indicated not only the heterogeneity in consumer preferences but also a potential source thereof.

Most of the standard deviation estimates of the Cholesky matrix were statistically significant in model 3, confirming that emotional eating style can explain heterogeneous preferences for nutritional claims presented on toast packages.

The results of model 3 are displayed below. The alternative specific constant (ASC) or no-buy option was found to be negative and significant at 1%, indicating that consumers obtain more utility from either of the two toast options than no purchase. Following economic theory, PRICE coefficient was negative and statistically significant, which means that consumers lose utility in proportion to the price increase of the alternatives. All estimated coefficients related to nutritional claims were positive, but only FAT and SALT were non-zero at 1%, suggesting that consumers perceive more utility from reduced-fat and low-salt toast—in this order—than conventional toast. The non-statistical significance of the FSALT coefficient indicates that they do not perceive any utility in toast packages bearing both nutritional claims and hence prefer the original product.

The derived standard deviations for all three parameters were non-zero at 1% significance, confirming that utility function differs among individuals; therefore, consumer preferences regarding nutritional claims are heterogeneous. All interaction term coefficients were statistically significant at 1% when the nutritional claim interacted with emotional eating style. This suggests that emotional eating style is a source of the preference heterogeneity. In particular, the interactions between the reduced-fat and the low-salt content claims with EE style (FAT × EE and SALT × EE, respectively) were negative, indicating that utility to an emotional eater decreases when the toasted bread presents only one nutritional claim (either reduced-fat or low-salt content by equal proportions).

Finally, following the procedure in Bazzani et al. [72], marginal WTP for reduced-fat and low-salt content claims at the mean values of the emotional eating style were calculated. Marginal WTP was obtained as a negative ratio of the mean value coefficient of the nutritional claims to the coefficient of the PRICE variable (FAT/PRICE, SALT/PRICE). The marginal WTP of the nutritional claim depended on the interaction with EE style; it was calculated as a negative ratio of the sum of the mean value coefficient of the nutritional claim and that of the interaction term divided by the PRICE coefficient (−(FAT + FAT × EE) ÷ PRICE).

Table 4 reports the calculated mean values of WTP for model 3. The WALD test revealed that only marginal WTP for reduced-fat and low-salt content was non-zero at 1% statistical significance. In particular, consumers were willing to pay €0.42 for reduced-fat toast and €0.25 for low-salt toast. However, when the WTP of the interaction term was compared, decreases of 9.52% and 16% on WTP for reduced-fat and low-salt claims, respectively, were observed. This result suggests that emotional eating style negatively impacts purchasing behavior.

## 4. Discussion and Conclusions

Today, food choices remain a hot topic in research on both eating and purchasing behavior, especially in the context of poor public health and high prevalence of non-communicable diseases. However, despite all efforts from policymakers to help citizens improve their diet, results are yet to be seen. Several behavioral theories can explain consumer preferences for healthier food products (i.e., those with nutritional claims). Jacquier et al. [74] and Bublitz et al. [75] highlighted that, to improve the effectiveness of public health policies, it is important to include other factors such as emotions in the analysis of consumers’ decision-making process. In this regard, the present study analyzes consumer preferences regarding toasted bread with nutritional claims and assesses if emotional eating style, as a coping strategy, influences purchase of this product.

The present results agree partially with previous empirical evidence. Cavaliere et al. [8], de-Magistris and López-Galán [10], Jurado and Gracia [12], and de-Magistris et al. [15] demonstrated that consumers show particular interest in reduced-fat claims. In fact, buyers perceive higher utility from reduced-fat toast than from other varieties. However, although preference for low-salt claims were positive, this effect is minor compared to reduced-fat claims; this finding supports Cavaliere et al. [8], who found that only 26% of their sample displayed strong interest in this nutritional claim. Conversely, de-Magistris and López-Galán [10] reported that Spanish consumers prefer conventional cheese over varieties with low-salt claims.

Additionally, de-Magistris et al. [15] noted heterogeneous preferences regarding low-salt potato chips. While pooled consumer samples appear to prefer low-salt claims, in fact, only obese individuals display a positive preference for this reformulated potato chip. When consumers consider the utility gained from toast carrying two nutritional claims, they do not show an interest in this type of toast. This finding contradicts de-Magistris et al. [10], who found that Spanish consumers prefer cheese bearing both reduced-fat and low salt claims. It also contrasts with de-Magistris et al.’s [15] findings that Spanish consumers perceive higher utility in potato chips bearing these two nutritional claims compared to the conventional variety. This discrepancy can be explained in part by the types of food product under investigation. Perhaps toasted bread is perceived by consumers to be healthier than cheese or potato chips. Another reason could be low market exposure to this type of nutritional claim, as only 6% of the food products on the Spanish market carry any nutritional claims related to salt content [76].

On the other hand, this study was the first to consider emotional eating as an influencing factor in the purchase of food products with nutritional claims. It identified whether emotions, manifesting as emotional eating, could represent an obstacle to effective public health policies (e.g., nutritional claims) and pose a challenge for policymakers in improving the food-related decisions of consumers.

In this respect, the mean EE scale rating and the frequency of the categories revealed that the participant sample presented an emotional eating style. Additionally, the analysis of consumer preferences suggested that emotional eating style negatively impacts purchasing behavior, as the WTP of the nutritional claims decreased between 9% and 16% for every unit increase in emotional eating. This finding supports the theory of emotional eating developed in the field of eating behavior, which states that negative emotions trigger high consumption of unhealthy foods such as energy-dense and sweet items [29,30,31,32,39].

In terms of the impact size, the psychological factor only explains between one-sixth and one-tenth of the heterogeneity in purchasing behavior seen in this study. This low explanatory power is due in part to the large percentage of participants who scored between 11 and 20 points on the EE scale (50%). These individuals know how to manage their emotions, especially negative ones. In some cases, they can be influenced by certain moods, but they have control over their eating behavior most of the time [67]. Thus, it is logical to assume that, when the harmful eating pattern (measured by the EE scale) is not clearly evident, the impact size on purchase decision will be minor.

While most studies have related emotional eating to consumption of energy-dense and sweet items, a salty food product was used in this analysis, which demonstrated the negative influence of this eating pattern on purchasing behavior. This result can be explained in part because bread is one of the first foods cut down when an individual begins a diet [58]. Furthermore, researchers such as Roberts [23] have found that some salty food products also motivate less healthy choices (e.g., emotional eating).

This study faced some limitations that offer insights for future research. First, the EEQ used considered only negative emotions as a stressor of emotional eating behavior. Hence, the impact of positive moods on purchasing behavior was not tested in the study. It would also be interesting to assess emotional influence on purchases conducted in a naturalistic environment, adopting other methodologies such as the ecological momentary assessment (EMA) to register behaviors, moods, and experiences of consumers in real time and in daily life. Second, because this study explored a non-typical energy-dense food product, future research could include a typical energy-dense food along with a neutral one to test if the product type moderates the impact of emotional eating style on purchasing behavior.

Finally, behavioral, political, and managerial implications may be derived from the present findings. First, the study highlighted that decision-making processes must be studied in a broader perspective, one that includes both rational and unconscious approaches—although this poses the challenge of developing new methodologies and measures for this purpose. Second, emotions—and the ability to manage them—should be considered by policymakers in designing public health policies that emphasize the pleasures associated with healthy eating habits. In this sense, outreach activities could be a key to reconditioning the public that sweets and other palatable foods are not the only ways to “comfort the soul”. Third, companies (e.g., supermarkets and food producers) must be more ambitious in their commitment to proposing more reformulated product (e.g., those with nutritional claims). They may even encourage consumers to choose items bearing less preferred nutritional claims with some promotional strategies.

The solution to public health intervention is undoubtedly complex and requires the engagement of society, the public, and the private sector as a whole.

## Figures and Tables

**Table 1:** Attributes and levels used in the choice experiment (CE) design.

Attributes	Levels
PRICE	€0.70
	€1.15
	€1.60
	€2.05
Reduced-fat claim (FAT)	0 = No label 1 = The amount of fat is reduced by 30% compared to traditional toast
Low-salt content (SALT)	0= No label 1 = The amount of salt in the toast is no more than 0.03 g per 100 g of product

**Table 2:** Sample sociodemographic characteristics.

Definition Variable	Population	Sample/(n = 306)
Sex		
Male	42.7	40.5
Female	50.9	59.6 ***
Age; mean (standard deviation)	42.9	45.4 (16.6)
18 to 34 years	24.1	27.8
35 to 55 years	39.2	40.9
More than 55 years	36.7	31.4
Education Level		
Primary studies	17.0	19.9
Secondary studies	50.0	43.5
University studies	33.0	36.6
Household Income		
Below €1500	N/A	32.2
Between €1501 and €2500	N/A	38.8
More than €2500	N/A	29.0
Body Mass Index; mean (standard deviation)		26 (4.4)
Normal weight (Below 25 kg/m^2^)	47.4	47.7
Overweight (Between 25 and 29.99 kg/m^2^)	35.7	33.0
Obesity (More than 30 kg/m^2^)	16.9	19.3
Emotional eating; mean (standard deviation)		12.6 (6)
Non-emotional eater *(score between 0–5)*		15.0
Low emotional eater *(score between 6–10)*		27.1
Emotional eater *(score between 11–20)*		50.0
Very emotional eater *(score between 21–30)*		7.8

N/A, Not Available; kg/m^2^, kilograms/square meters; ***, **, * indicate significance at 1%, 5%, and 10% level.

**Table 3:** Parameter estimates from the parameter logit model (RPL) and the parameter logit model with error content (RPL-EC) models.

	Model 1	Model 2	Model 3
Variable Coefficients	(RPL)	(RPL-EC)	(RPL-EC with EE interactions)
FAT	0.52 (0.18) ***	0.94 (0.22) ***	1.28 (0.22) ***
SALT	0.01 (0.16)	0.36 (0.21) *	0.77 (0.24) ***
FSALT	1.06 (0.21) ***	0.36 (0.27)	0.11 (0.25)
PRICE	−2.46 (0.13) ***	−3.11 (0.12) ***	−3.07 (0.12) ***
ASC	−4.14 (0.24) ***	−5.70 (0.31) ***	−6.02 (0.33) ***
Error component		3.68 (0.18) ***	3.69 (0.18) ***
Interaction terms with EE			
FAT × EE			−0.11 (0.22) ***
SALT × EE			−0.11 (0.03) **
FSALT × EE			0.10 (0.03) ***
No. of parameters	8	12	12
Log likelihood (LL)	−2536.30	−2231.15	−2222.53
AIC	5088.6	4486.3	4475.1
AIC/N	1.39	1.22	1.22

***, **, * indicate significance at 1%, 5%, and 10% level. FAT= reduced-fat; SALT= low-salt content; FSALT= an interaction between both reduced-fat and low-salt content; EE= emotional eating; ASC= alternative specific constant; AIC: Akaike information criterion.

**Table 4:** Marginal willingness to pay (WTP) for toasted bread with reduced-fat and low-salt content claims.

	Marginal WTP	Standard Error
FAT ***	€0.42	0.08
SALT ***	€0.25	0.08
FAT × EE ***	€0.38	0.08
SALT × EE ***	€0.21	0.08

***, **, * indicate significance at 1%, 5% and 10% level.

## Appendix A

**Table A1:** Cuestionario del Comerdor Emocional Garaulet (CCE).

¿La báscula, tiene un gran poder sobre ti? ¿Es capaz de cambiar tu estado de humor?			
□	□	□	□
Nunca	A veces	Generalmente	Siempre
¿Tienes antojos por ciertos alimentos específicos?			
□	□	□	□
Nunca	A veces	Generalmente	Siempre
¿Te cuesta parar de comer alimentos dulces, especialmente chocolate?			
□	□	□	□
Nunca	A veces	Generalmente	Siempre
¿Tienes problemas para controlar las cantidades de ciertos alimentos?			
□	□	□	□
Nunca	A veces	Generalmente	Siempre
¿Comes cuando estás estresado, enfadado o aburrido?			
□	□	□	□
Nunca	A veces	Generalmente	Siempre
¿Comes más de tus alimentos favoritos, y con más descontrol, cuando estás solo?			
□	□	□	□
Nunca	A veces	Generalmente	Siempre
¿Te sientes culpable cuando tomas alimentos “prohibidos”, es decir, aquellos que crees que no deberías, como los dulces o snacks?			
□	□	□	□
Nunca	A veces	Generalmente	Siempre
Por la noche, cuando llegas a casa cansado de trabajar ¿es cuando menos control tienes con tu alimentación?			
□	□	□	□
Nunca	A veces	Generalmente	Siempre
Estás a dieta, y por alguna razón comes más de la cuenta, entonces piensas que no vale la pena y ¿comes de forma descontrolada aquellos alimentos que piensas que más te van a engordar?			
□	□	□	□
Nunca	A veces	Generalmente	Siempre
¿Cuántas veces sientes que la comida te controla a ti en vez de tú a ella?			
□	□	□	□
Nunca	A veces	Generalmente	Siempre

**Table A2:** Emotional Eater Questionnaire (EEQ) Garaulet.

Do the weight scales have a great power over you? Can they change your mood?			
□ Never	□ Sometimes	□ Generally	□ Always
Do you crave specific foods?			
□ Never	□ Sometimes	□ Generally	□ Always
Is it difficult for you to stop eating sweet things, especially chocolate?			
□ Never	□ Sometimes	□ Generally	□ Always
Do you have problems controlling the amount of certain types of food you eat?			
□ Never	□ Sometimes	□ Generally	□ Always
Do you eat when you are stressed, angry or bored?			
□ Never	□ Sometimes	□ Generally	□ Always
Do you eat more of your favorite food and with less control when are alone?			
□ Never	□ Sometimes	□ Generally	□ Always
Do you feel guilty when eat “forbidden” foods, like sweet or snacks?			
□ Never	□ Sometimes	□ Generally	□ Always
Do you feel less control over your diet when you are tired after work at night?			
□ Never	□ Sometimes	□ Generally	□ Always
When you overeat while on a diet, do you give up and start eating without control, particularly food that you think is fattening?			
□ Never	□ Sometimes	□ Generally	□ Always
How often do you fell that food controls you, rather than you controlling food?			
□ Never	□ Sometimes	□ Generally	□ Always
